# Late gadolinium enhancement imaging and sudden cardiac death

**DOI:** 10.1093/eurheartj/ehaf464

**Published:** 2025-07-16

**Authors:** Sanjay K Prasad, Tamim Akbari, Martin J Bishop, Brian P Halliday, Francisco Leyva-Leon, Francis Marchlinski

**Affiliations:** Royal Brompton and Harefield Hospitals, Part of Guy’s and St Thomas’ NHS Foundation Trust, CMR Unit, Sydney Street, London SW3 6NP, UK; Imperial College London, National Heart and Lung Institute, Guy Scadding Building, Cale Street, London SW3 6LY, UK; Royal Brompton and Harefield Hospitals, Part of Guy’s and St Thomas’ NHS Foundation Trust, CMR Unit, Sydney Street, London SW3 6NP, UK; Imperial College London, National Heart and Lung Institute, Guy Scadding Building, Cale Street, London SW3 6LY, UK; School of Biomedical Engineering and Imaging Sciences, King’s College London, Lambeth Wing St. Thomas' Hospital, Westminster Bridge Road, London SE1 7EH, UK; Royal Brompton and Harefield Hospitals, Part of Guy’s and St Thomas’ NHS Foundation Trust, CMR Unit, Sydney Street, London SW3 6NP, UK; Imperial College London, National Heart and Lung Institute, Guy Scadding Building, Cale Street, London SW3 6LY, UK; Aston Medical School, Translational Medicine Research Group (TMRG) College of Health and Life Sciences, Aston University, Birmingham B4 7ET, UK; Penn Heart and Vascular Center, Hospital of the University of Pennsylvania, Philadelphia, PA, USA

**Keywords:** Sudden cardiac death, Late gadolinium enhancement, Cardiac magnetic resonance, Cardiomyopathy, Outcomes

## Abstract

The prediction and management of sudden cardiac death risk continue to pose significant challenges in cardiovascular care despite advances in therapies over the last two decades. Late gadolinium enhancement (LGE) on cardiac magnetic resonance—a marker of myocardial fibrosis—is a powerful non-invasive tool with the potential to aid the prediction of sudden death and direct the use of preventative therapies in several cardiovascular conditions. In this state-of-the-art review, we provide a critical appraisal of the current evidence base underpinning the utility of LGE in both ischaemic and non-ischaemic cardiomyopathies together with a focus on future perspectives and the role for machine learning and digital twin technologies.

## Graphical Abstract

**Graphical Abstract:**
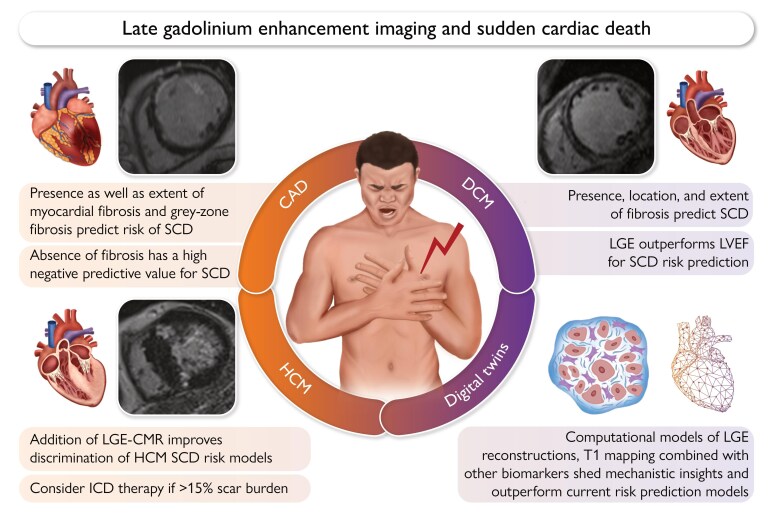
Late gadolinium enhancement cardiac magnetic resonance and sudden cardiac death in cardiomyopathies. *CAD*, coronary artery disease; *CMR*, cardiac magnetic resonance; *DCM*, dilated cardiomyopathy; *HCM*, hypertrophic cardiomyopathy; *ICD*, implantable cardioverter defibrillator; *LGE*, late gadolinium enhancement; *LVEF*, left ventricular ejection fraction; *SCD*, sudden cardiac death.

## Introduction: sudden cardiac death and fibrosis

Sudden cardiac death (SCD) affects around 50 to 100 per 100 000 of the population and accounts for up to 50% of all cardiovascular deaths.^1,2^ Despite a decline in incidence of SCD, the identification and prevention of SCD continues to present a significant challenge with as yet no clear strategy for mass screening in the general population.^3,4^ Several markers have been shown to associate with the risk of sudden death in cardiovascular disease. Whilst not validated in whole population screening, late gadolinium enhancement (LGE) cardiac magnetic resonance (CMR) for characterizing fibrosis, is one powerful imaging technique shown to independently predict the risk of SCD in cardiovascular disease (*Graphical Abstract*). In this article, we present a state-of-the-art review of the evidence underpinning the role of LGE in SCD prediction in various cardiovascular disease states.

### Fibrosis and late gadolinium enhancement

A characteristic pathological feature often seen in patients with cardiac disease is the presence of myocardial fibrosis which results from an increase in collagen formation in the extracellular matrix and myocyte cell death. Three forms of fibrosis have been identified on histopathological analysis. Replacement fibrosis represents areas of myocardial scarring because of myocyte cell death whereas interstitial fibrosis represents a net accumulation of extracellular matrix proteins in the absence of significant cardiomyocyte loss. A third type known as perivascular fibrosis describes the expansion of the microvascular adventitia.^5^ The activation of the renin–angiotensin–aldosterone system and the beta-adrenergic axis in heart failure syndromes, myocardial injury, and various genetic and environmental insults lead to the activation of the inflammatory cascade resulting in myofibroblast activation, production of collagen and myocyte cell death.^5,6^ The resultant fibrosis and the grey-zone surrounding areas of fibrosis containing a heterogeneous mixture of viable and non-viable myocardium are thought to provide the substrate for ventricular arrhythmia through abnormal automaticity, triggered activity, and re-entry.^7–10^

LGE on CMR affords the ability to non-invasively identify areas of cardiac fibrosis with high spatial resolution and serves as the reference standard for myocardial tissue characterization.^11^ Since its development in the 1980s, LGE-CMR has become the gold standard in the non-invasive diagnosis of a range of ischaemic and non-ischaemic cardiomyopathies.^12^ Numerous studies and meta-analyses have underlined the clinical significance and prognostic ability of LGE as a powerful predictor of SCD and ventricular arrhythmia.^13–16^

## Sudden cardiac death and late gadolinium enhancement in coronary artery disease

Coronary artery disease (CAD) remains the largest cause for SCD accounting for more than 75% of the cases.^17–19^ Guidelines for primary prevention implantable cardioverter-defibrillators (ICD) recommend using a left ventricular ejection fraction (LVEF) cut-off value of 35% or less for ischaemic aetiology with a class IA recommendation^2,20^ based on historical trials conducted over two decades ago^21–24^ and supported by more recent registry data.^25^ This method has several limitations. First, in terms of absolute numbers, more patients with mild or moderate left ventricular (LV) impairment suffer SCD as compared with patients with severe LV impairment.^26^ Second, severe LV impairment is a risk factor for both sudden and non-sudden death.^27^ Third, only a small proportion of patients with primary prevention ICD ever need therapy,^28^ therefore, requiring high numbers needed to treat to prevent one sudden death and presenting the challenges of device complications, reintervention, infection, economic cost and psychological burden of inappropriate shocks. In a large pooled analysis of 20 data sets comprising of 140 204 patients post-myocardial infarction by the PROFID (Prevention of SCD after myocardial infarction by defibrillator implantation) group, LVEF was shown to be a poor predictor of sudden death, reporting an area under the receiver operating characteristic curve between 0.50 and 0.56.^29^

A number of studies have shown fibrosis detected by LGE on CMR to be a better predictor of sudden death in ischaemic cardiomyopathy when compared with LVEF (*Table 1*).^30–34^ In a large study by Zegard *et al.* (979 patients with CAD, 29 SCDs, 80 arrhythmic endpoints; average follow-up 5.8 years), both myocardial fibrosis (hazard ratio [HR] 10.1, 95% confidence interval [CI] 1.42–1278.9) as well as grey-zone fibrosis (using 3 standard deviation [SD] method > 5.0 g, sub-distribution HR 10.8; 95% CI 3.74–30.9) were better predictors of SCD as compared with LVEF (LVEF <35% and SCD; sub-distribution HR 2.99, 95% CI 1.42–6.31). In addition, the absence of myocardial fibrosis had high negative predictive value for SCD and arrhythmic endpoints approaching 100% sensitivity.^32^

**Table 1:** Studies of LGE and SCD in ICM

Study	*N*	Design	Follow-up, years (median/mean)	Aetiology	Results	Conclusions
Jones *et al*.^30^	437	Single centre, Prospective registry	6.3	ICM	LGE mass (per gram) and SCD/aSCD PIZ HR 1.07 (95% CI 1.02–1.12; *P =* .002) Core infarct HR: 1.03 (95% CI 1.01–1.05; *P =* .01)	PIZ mass and core infarct mass were independently associated with the primary outcome
Pontone *et al*.^31^	861	Multi-centre registry	12.8	ICM	LGE mass and MAACE (composite SCD/aSCD/sustained VT) HR 1.010 (95% CI 1.002–1.018; *P* = .015)	LGE mass was one of the independent predictors of MAACE
Leyva *et al*.^33^	700	Single centre, prospective registry of patients undergoing CIED implants	6.93	ICM (58.3%) and NIDCM (41.7%)	MF and SCD HR 26.3 (95% CI 3.7–3337) NPV: 100% GZF_mass_ and SCD HR 44.6 (95% CI 6.12–5685)	In CIED recipients, MF and GZF_5SD_ mass were strong predictors in relation to SCD and the arrhythmic endpoint
Zegard *et al*.^32^	979	Single centre, retrospective registry	5.82	ICM	MF and SCD HR 10.1 (95% CI 1.42–1278.9)	MF on visual assessment and quantified GZF_3SD_ mass were strongly associated with SCD and VAs
Haghbayan *et al*.^35^	1518	Meta-analysis of 20 studies	3.6	ICM	PIZ and appropriate ICD therapy (5 studies; *n* = 361) HR 1.31/10 g (95% CI 1.17–1.47)	Quantification of the PIZ predicted long-term mortality and appropriate ICD therapy
Klem *et al*.^34^	137	Single centre, prospective	2	ICM	Scar size >5% and death/appropriate ICD discharge for sustained VT HR 5.2 (95% CI 2.0–13.3)	Scar was an independent predictor of adverse outcomes

aSCD, aborted sudden cardiac death; CI, confidence interval; CIED, cardiac implantable electronic device; GZF, grey-zone fibrosis; HR, hazard ratio; ICD, implantable cardioverter defibrillator; ICM, ischaemic cardiomyopathy; LGE, late gadolinium enhancement; LVEF, left ventricular ejection fraction; MAACE, major arrhythmic adverse cardiovascular event; MF, myocardial fibrosis; NIDCM, non-ischaemic dilated cardiomyopathy; NPV, negative predictive value; PIZ, peri-infarct zone; SCD, sudden cardiac death; SD, standard deviation; VA, ventricular arrhythmia; VT, ventricular tachycardia.

There is also evidence that quantification of core and grey-zone or peri-infarct zone fibrosis in CAD are independently associated with SCD and overall mortality.^35–37^ In a study by Jones *et al.*, in 437 patients with stable CAD (median follow-up 6.3 years, 49 SCD, or aborted SCD events) who underwent comprehensive CMR, incrementally, core infarct mass and grey-zone mass were independently associated with SCD or aborted SCD (per gram HR 1.07, 95% CI 1.02–1.12; *P* = .002).^30^

### ICD implants in CAD

Although LGE on CMR is emerging as a strong predictor of outcomes, many factors contribute to the risk of sudden death in CAD and no one parameter is likely to capture all risk. This is further complicated by the observation that patients with ischaemic heart disease are likely to be older with multiple other comorbidities and therefore have competing risk of death from non-sudden causes. The decision for ICD implant in these patients needs to move away from dichotomous LVEF measurements and incorporate other prognostic variables. Younis *et al*. have developed a risk score (MADIT-ICD benefit score) based on data from 4531 patients (two-thirds with ischaemic cardiomyopathy; mean LVEF 25 ± 6%) enrolled in the MADIT trials. In their analysis, the 3-year predicted risk of ventricular tachycardia (VT)/ventricular fibrillation was three-fold higher than the risk of non-arrhythmic mortality (20% vs. 7%, *P* < .001) in the highest benefit group.^38^ This probabilistic analysis has limitations which include retrospective analysis of historical trial data (rather than real world, community level data), lack of availability of LGE or genetic factors and lack of independent external validation. The evidence base upon which ICD recommendations are prescribed in the guidelines is over 20 years old and currently, risk prediction models are not in clinical use due to lack of robust prospective external validation in a randomized controlled trial setting.

## Sudden cardiac death, LGE, and hypertrophic cardiomyopathy

Hypertrophic cardiomyopathy (HCM), defined as the presence of increased LV wall thickness or mass not explained by abnormal loading conditions, has a prevalence of around 1 in 500 and is one of the leading causes of sudden death in young adults.^39–42^ In about 30%–40% of cases, a sarcomeric genetic variant is identified.^43^ The annual incidence of sudden death and aborted sudden death is reported to be around 0.8% but this varies widely depending on risk profile.^2,44–46^ Multiple factors have been implicated in pathophysiology of arrhythmogenesis in HCM. Markedly hypertrophied regions correspond to disorganized cardiomyocyte architecture with fibrosis and collagen matrix deposition on histological studies.^47^ These changes can lead to local conduction delay or block, abnormally fractionated and prolonged endocardial bipolar electrograms with reduced voltage amplitudes.^48^ Ischaemia both due to remodelled intramural coronary arteries^49,50^ and microvascular dysfunction^51^ as well as abnormal handling of calcium homeostasis in pre-clinical studies have also been implicated.^52,53^

Identification of individuals at risk of sudden death who would most benefit from primary ICD is challenging. A 5-year SCD risk stratification score based on seven factors (age, LV wall thickness, left atrial size, LV outflow tract gradient, non-sustained VT, unexplained syncope, and family history of SCD) was developed^54^ and has been externally validated.^55,56^ A 5-year SCD score of 6% or more is considered high risk leading to a class IIa recommendation for ICD implantation whereas a score of <4% is considered low risk and in between (≥4% to <6%) considered intermediate risk. Given the emerging role of imaging in SCD risk stratification, some data suggest that this risk scoring based on clinical parameters alone lacks sufficient discriminatory power.^57,58^ LGE is seen in about 60% of patients with a confirmed HCM diagnosis. A large number of HCM studies and their meta-analyses have shown a strong association of fibrosis as assessed by LGE on CMR and malignant ventricular arrhythmia, sudden death, and aborted sudden death (*Table 2*), suggesting LGE assessment to be an important additive parameter in prognostic modelling when assessing SCD risk and preventive ICD therapies.^60–67^ These findings also extend to the adolescent age group.^68^ In a multi-centre study of 493 patients (23 events; median follow-up 3.4 years), LGE on CMR outperformed both ESC HCM Risk-SCD score and the ACCF/AHA criteria (C-statistic 0.84, 95% CI 0.76–0.91).^69^ Moreover, in a recent pooled meta-analysis of eight observational studies (*n* = 4519), the absence of LGE was associated with a low annual risk of SCD (0.34%/year) equating to 80% lower risk as compared with LGE positive patients over a 10 year follow-up period.^59^ Of note, although there is evidence to suggest a strong negative predictive value, absence of LGE does not preclude the risk of SCD.

**Table 2:** Meta-analyses of LGE and SCD in HCM

	*N*	No. of studies	Follow-up, years (median/mean)	Results	Conclusion
Abdelfattah *et al*.^59^	4519	8	3.4	SCD (LGE +) HR 5.00 (95% CI 3.21–7.78; *P* < .001)	The absence of LGE was associated with a low annual risk for SCD events
Kiaos *et al*.^60^	5550	11	5.2	LGE extent and SCD pooled OR 4.93 (95% CI 3.75–6.47)	All quantification techniques were comparable. With six SD technique, LGE 10% was the optimal cut-off to effectively re-stratify intermediate-risk patients
Kamp *et al*.^61^	3808	8	3.2	LGE and: SCD OR 1.69 (95% CI 1.03–2.78) SCD or aborted SCD OR 2.32 (95% CI 1.56–3.43)	LGE on CMR was a strong predictor of arrhythmic outcomes including SCD, aborted SCD, and appropriate ICD therapy.
Fortuni *et al*.^62^	3351	7	2.97	LGE and: SCD/aborted SCD OR 3 .34 (95% CI 1.97–5.69; *P* < .001)	Presence of LGE at CMR in patients with HCM had a substantial prognostic value for fatal events and, in particular, for SCD.
He *et al*.^63^	3770	9	2.9	SCD/aborted SCD in LGE (+) vs. LGE (−) OR 3.40 (95% CI 1.90–6.08; *P* < .001)	LGE was significantly associated with SCD/aborted SCD risk in patients with HCM.
Weng *et al*.^64^	2993	5	3.1	LGE and SCD OR 3.41 (95% CI 1.97–5.94; *P* < .001) Extent of LGE and SCD HR 1.56/10% LGE (95% CI 1.33–1.82; *P* < .0001)	Quantitative LGE by CMR exhibited a substantial prognostic value in SCD events prediction, independent of baseline characteristics
Briasoulis *et al*.^65^	3067	6	3.05	SCD and LGE (+) vs. LGE (−) OR 2.52 (95% CI 1.44–4.4; *P* = .001)	LGE was significantly associated with SCD risk
Green *et al*.^66^	1063	4	3.1	LGE and SCD/aborted SCD pooled OR 2.39 (95% CI 0.87–6.58; *P* = .091)	LGE was associated with increased risk of SCD/aSCD

CI, confidence interval; CMR, cardiac magnetic resonance; HCM, hypertrophic cardiomyopathy; HR, hazard ratio; ICD, implantable cardioverter-defibrillator; LGE, late gadolinium enhancement; OR, odds ratio; SCD, sudden cardiac death; SD, standard deviation.

### LGE quantification and SCD in hypertrophic cardiomyopathy

Quantification and not merely the binary presence of LGE has also been shown in a number of studies to portend prognostic value.^60,67,69,70^ In a study of low and intermediate risk patients (*n* = 1423; SCD/aborted SCD events 60 [4%]; mean follow-up 4.7 years), on quadratic spline analysis, LGE ≥15% was associated with increased risk of composite events and addition of LGE ≥15% to the ESC 5-year SCD risk score improved the log likelihood ratios from −227.85 to −219.14 (χ^2^ 17).^71^ There is some debate about the utility of quantitative LGE on CMR as no consensus exists on the optimal quantification method. In a recent meta-analysis of 11 studies of quantitative LGE in HCM (*n* = 5550, median follow-up 5.2 years; consisting of 4 methods of quantification) all methods had comparable accuracy in predicting SCD and LGE 10% cut-off using 6 SD technique was able to risk stratify in intermediate cases (sensitivity 0.73 and specificity 0.67).^60^ Acknowledging the key role of LGE in risk stratification, both the latest European and American guidelines recommend considering ICD therapy in patients with LGE of ≥15% (ESC) or ‘extensive’ LGE (ACCF/AHA), especially in borderline or intermediate risk cases.^2,72^ The cut-off value of 15% has been questioned by recent studies as thresholds of 5% and 10% have also been shown to associate with higher SCD events as compared with LGE below 5% after multi-variable adjustment.^60,73^ Additional factors of interest in predicting sudden death in this patient population include LV apical aneurysms, LV systolic dysfunction and presence of sarcomeric mutations.^2^

LGE on CMR is emerging as a powerful tool in aiding the decision for ICD therapy in patients with HCM, in particular those that fall in the more clinically challenging low and intermediate risk categories. However, given the heterogeneous nature of the disease, the use of multiple variables reflecting different aspects of the disease may be necessary to provide an accurate estimate of prognosis. Furthermore, the use of LGE to guide ICD therapy in HCM needs to be evaluated prospectively in a randomized controlled trial.^74^

## LGE and SCD in dilated cardiomyopathy

Dilated cardiomyopathy (DCM) is characterized by LV dilatation and reduced systolic function in the absence of coronary disease or abnormal loading conditions. The true prevalence of DCM is likely underestimated and is thought to be around 1 in 250^75^ making it one of the most common cardiomyopathies, carrying a 20% 5-year mortality.^76,77^ The reported incidence of SCD in DCM varies ranging from 0.1% to 4% annuallly.^77–79^

Guidelines currently recommend ICDs to reduce the risk of SCD in symptomatic DCM patients (New York Heart Association class II-III) with a LVEF <35% (class IIa recommendation).^2,42^ Evidence for LVEF based stratification comes from meta-analysis of the five trials that have evaluated ICD therapy in patients with DCM and severely impaired LV function.^80,81^ In the DANISH trial, the largest trial including patients with DCM investigating ICD therapy vs. optical medical therapy, all-cause mortality was not lower in patients with ICDs (HR 0.87, 95% CI 0.68–1.12; *P* = .28); however, SCD was reduced (HR 0.50, 95% CI 0.31–0.82; *P* = .005).^82^ This discrepancy might be explained in part by the competing risk from non-sudden causes of death.^83,84^

A number of studies have shown that LVEF-based risk stratification for ICD implantation is imprecise as the majority of devices implanted never need to deliver therapy (11.5% appropriate shock in DANISH trial over 5.6-year follow-up)^82^ and a cohort of patients with a LVEF >35% go on to suffer SCD.^79,85^

### Extent and location of LGE in DCM

Characteristic mid-wall LGE occurs in up to one third of DCM patients. Both the presence and specific location of LGE act as substrates for ventricular arrhythmias associating with a five- to nine-fold increased risk of SCD.^79,86–89^ Septal LGE carries a higher risk than lateral wall fibrosis alone.^89^ In patients with DCM and an LVEF ≥40%, mid-wall LGE identifies a subset at increased risk of SCD and a low risk of non-sudden death (HR 4.8, 95% CI 1.7–13.8; *P* = .003).^88^ Even in patients with normal LV function by echocardiogram and frequent pre-mature ventricular contractions, a non-ischaemic ring-like mid-wall, LV scar pattern predicted more than twice the risk of a composite of death and major arrhythmic events during follow-up compared with those patients with a non-ring like LGE pattern likely to reflect a higher prevalence of high-risk genetic phenotypes.^90^ In patients with sustained VT in the setting of DCM, despite a diffuse decrease in LV function, the areas of replacement fibrosis and in turn the regions of LGE on magnetic resonance imaging that correlate with the VT substrate during electroanatomic mapping are located predominantly in the basal and typically perivalvular septum and/or LV free wall.^91–93^

Similar to the role of LGE quantification in HCM, there is emerging evidence for its prognostic role in DCM. In a recent study of two large UK tertiary centres, using competing risk analyses, quantification of both total fibrosis and grey-zone fibrosis in patients with DCM added incremental value in predicting the risk of SCD or ventricular arrhythmia. Total fibrosis mass of >10 g was associated with the highest risk (HR 9.17, 95% CI 4.64–18.1) compared with patients with no visual fibrosis.^94^

Multiple meta-analyses have shown LGE on CMR to associate with the risk of SCD in DCM (*Table 3*).^14,16,95–100^ In the most up to date and largest meta-analysis of 103 studies (*n* = 29 687) LGE presence and extent (per 1%) were associated with higher arrhythmic endpoints (HR 2.69, 95% CI 2.20–3.30; *P* < .001 and HR 1.07, 95% CI 1.03–1.12; *P* = .004) in addition to associating with higher all-cause mortality, cardiovascular mortality, and heart failure events. On the contrary, LVEF did not predict mortality or arrhythmic endpoints.^95^

**Table 3:** Meta-analyses of LGE and clinical outcomes in DCM

Study	*N*	No. of studies	Follow-up, years (median/mean)	Results	Conclusion
Eichhorn *et al*.^95^	29 687	103	3.1	LGE and VA: HR 2.69 (95% CI 2.20–3.30; *P* < .001)	Presence and extent of LGE were associated with arrhythmic endpoints in NICM
Theerasu-wipakorn *et al*.^14^	15 217	60	3	LGE and VA pooled OR: 3.99 (95% CI 3.08–5.16)	Real-world evidence suggested that the presence of LGE on CMR was a strong predictor of adverse long-term outcomes in patients with NICM
Di Marco *et al*.^16^	2948	29	3	Weighted rate difference LGE (+) and LGE (−) 4% (95% CI 2.6% to 5.5%; *P* < .001) pooled OR 4.3 (*P* < .001)	The presence of LGE was associated with significantly higher occurrence of arrhythmic endpoints including in patients with LVEF > 35%
Wang *et al*.^96^	1827	7	3	Pooled OR of mid-wall fibrosis and: SCD/aborted SCD 2.25 (95% CI 1.16–3.16)	The presence of LV mid-wall fibrosis on LGE is a significant prognosticator of adverse events in NICM patients
Disertori *et al*.^97^	2850	19 (both ICM and NICM)	2.8	Overall population, LGE (+) vs. LGE (−) and VA Pooled OR 5.62 (95% CI 4.20–7.51)	LGE was a powerful predictor of ventricular arrhythmic risk in patients with ventricular dysfunction, irrespective of aetiology
Becker *et al*.^99^	4554	34	3	LGE (+) vs. LGE (−) VA OR 4.52 (95% CI 3.41–5.99)	The presence of LGE on CMR substantially worsens prognosis for adverse cardiovascular events in DCM patients
Kuruvilla *et al*.^100^	1488	9	2.5	LGE (+) vs. LGE (−) SCD/aborted SCD OR 5.32 (*P* < .00001)	LGE in patients with NICM is associated with increased risk of SCD

CMR, cardiac magnetic resonance; DCM, dilated cardiomyopathy; HR, hazard ratio; ICM, ischaemic cardiomyopathy; LGE, late gadolinium enhancement; NICM, non-ischaemic cardiomyopathy; OR, odds ratio; SCD, sudden cardiac death; VA, ventricular arrhythmia.

### Genetic profile and SCD in DCM

The genetic architecture also confers risk. A familial cause is implicated in about 20%–35% of DCM cases.^101^ Carriers of specific genetic variants associated with DCM, for instance desmosomal, lamin A/C, filamin C, and titin-truncating variants, carry an increased risk of SCD.^102,103^ This may in part be explained by the varying distribution pattern of LGE underlying the genetic substrate. In a recent large study of 577 patients with DCM across 20 Spanish centres by de Frutos *et al.* (causative genetic variant = 38%; LGE-positive 25.5%), patients with LGE had a higher genetic yield of pathogenic and likely pathogenic genetic variants (30%–50%) as compared with patients with no LGE (27.3%). At a median follow-up of 2.7 years, distinct patterns of LGE (sub-epicardial, mid-wall linear, transmural, and right ventricular insertion) were associated with higher risk of major ventricular arrhythmias (SCD or aborted SCD, sustained VT, and appropriate ICD interventions) as compared with no LGE, the highest risk group being those with a combination of these patterns (HR 18.2, 95% CI 5.1–64.4; *P* < .001).^104^ On the other hand, sub-endocardial and patchy LGE did not associate with major ventricular arrhythmia in this study. This along with other published literature suggests that the pattern of LGE has a significant prognostic role in DCM.^87,105^

### Risk stratification for ICD in DCM

There is a pressing need for improved risk stratification for SCD in DCM patients, to ensure ICDs are implanted in those most likely to benefit and avoided in others. This is important as ICDs are expensive. An economic analysis by National Institute for Health and Care Excellence, UK in the financial year 2011 reported a cost of £9692 per system^106^ whilst carrying a 5% risk of infection, a 2% risk of pneumothorax and a 6% risk of inappropriate shocks over 5 years with substantial impact on quality of life.^82^ Acknowledging these gaps, in the current era of improved outcomes secondary to modern guideline-directed heart failure therapy, the recently published cardiomyopathy guidelines from the ESC conclude that an optimized strategy for sudden death prevention in DCM remains unsolved highlighting the need for better risk stratification when offering ICD implantation.^42^

### LV ring-like LGE and SCD

In a subset of patients, on LGE-CMR, a ring-like scar pattern is seen with extensive mid-wall/sub-epicardial fibrosis affecting at least three contiguous segments in the same short-axis slice. Although it appears to correlate with a genetic cause for DCM and in particular with desmosomal and filamin C gene variants^105^ in about 15% of cases, an inflammatory cardiomyopathy is diagnosed.^107^ It is associated with an elevated risk of malignant arrhythmias. In a multi-centre study of 686 patients, those with ring-like scarring (4% of the cohort) had a 50% rate of adverse outcomes (death, cardiac arrest, or appropriate ICD therapy) over 5 years, compared with 19% in non-ring-like scarring and 0.3% in LGE-negative patients. After multi-variable adjustment, the presence of LGE with ring-like pattern remained independently associated with increased risk of the composite endpoint (HR 68.98, 95% CI 14.67–324.39; *P* < .01).^90^ Similarly, in another multi-centre study, ring-like LGE in 115 patients and the presences of at least one other high-risk feature (pathogenic/likely pathogenic genetic variant, family history for cardiomyopathy or arrhythmogenic cardiomyopathy diagnosis) was associated with a high burden of life-threatening arrhythmia (3.8 events/100 patients/year; median follow-up 4.6 years). On multi-variable analysis, anterior Q-waves, QRS width, and LV end-diastolic volume index were independently associated with life-threatening arrhythmias (Harrell’s C-index = 0.796).^108^

## LGE and other pathologies

LGE has implications in many other cardiac pathologies. A number of studies have shown a strong association between LGE and SCD in patients with valvular heart disease, in particular aortic stenosis (AS) and mitral valve prolapse (MVP) with mitral annular disjunction (MAD).

### LGE and aortic stenosis

The annual incidence of SCD in AS is reported to be around 1.8% per annum in symptomatic patients and 0.39% to 1.4% in asymptomatic individuals.^109,110^ Fibrosis as detected via LGE on CMR has been shown to precede symptom development indicating irreversible damage and strongly associates with mortality.^111–114^ In a recent meta-analysis of 13 studies (*n* = 2430; follow-up 6–67.2 months), LGE in patients with AS was also shown to associate with a composite outcome of major adverse cardiovascular events which included SCD (pooled relative risk 1.649, 95% CI 1.23–2.22, *P* = .001).^115^ The latest EVOLVED randomized trial of intervention in asymptomatic severe AS with myocardial fibrosis, there was no significant difference in the primary composite endpoint of all-cause death or unplanned AS–related hospitalization in patients randomized to receive early intervention vs. patients randomized to receive guideline-directed conservative management.^116^ Low event rates, lack of long-term follow-up and a paucity of data on SCD in patients with AS means no firm conclusions can be drawn on the prognostic relevance of LGE in risk stratification for SCD in patients with AS.

### LGE and mitral valve prolapse

MAD, a term which describes a distinct separation of the mitral valve annulus and left atrial wall continuum, often occurs in conjunction with MVP and has been shown to associate with ventricular arrhythmia and SCD.^117–119^ There is emerging evidence that LGE has a prognostic role in this cohort, in particular, myocardial fibrosis affecting both the infero-basal LV free wall and the papillary muscles has been recognized.^120^ Investigators from 15 tertiary European centres (*n* = 474; mean follow-up 3.3 years) reporting outcomes in patients with MVP found that LGE (HR 4.2; *P* = .006) and the extent of LGE (HR 1.2 per 1% increase; *P* = .006) predicted more adverse events (sustained VT, SCD, and unexplained syncope) as compared with MAD (*P* = .89).^121^ Moreover, LGE-positive patients are more likely to have longer MAD distance, which in itself is an adverse prognostic marker when it comes to malignant arrhythmias.^121–123^ Based on current evidence, LGE on CMR has been recognized as a potential factor in risk stratification of the ‘arrhythmic MVP syndrome’ in the latest guidelines.^2^

### LGE and sarcoidosis

Sarcoidosis, which is a multi-system inflammatory condition, can lead to fibrosis in the heart pre-disposing patients to malignant ventricular arrhythmias.^124^ LGE detection of fibrosis in cardiac sarcoidosis has been shown to strongly associate with ventricular arrhythmia in a number of studies and confirmed in a recent meta-analysis of 13 studies with the highest odds ratio of risk seen when biventricular scarring is noted.^125^ A separate meta-analysis suggests the presence of LGE is associated with a nine fold increased risk of life-threatening ventricular arrhythmias.^126^ Risk increases with biventricular disease.^127^ Current guidelines recommend an ICD for patients with cardiac sarcoidosis and ‘extensive’ LGE on CMR with a class IIa recommendation.^2^

Cardiac amyloidosis caused by misfolded precursor proteins leading to deposits in heart is mainly related to light-chain amyloid or transthyretin amyloid. A characteristic LGE pattern is seen in cardiac amyloidosis; typically, of global sub-endocardial LGE, coupled with abnormal myocardial and blood pool gadolinium kinetics.^128^ Mode of death in the disease is mostly progressive heart failure and there is uncertainty about the benefit of ICD therapy in this setting.^129^

### LGE and myocarditis

Myocarditis has an annual incidence of 4–14 per 100 000 people with variable consequences ranging from mild LV dysfunction to severe impairment, heart block and life-threatening arrhythmia.^130,131^ In up to 12% of SCD cases, myocarditis is implicated on autopsy.^130,132^ LGE plays a prognostic role when it comes to sudden death and major ventricular arrhythmia in myocarditis.^133^ In one study analysing 156 patients with a diagnosis of myocarditis and life-threatening arrhythmia, LGE (≥2 myocardial segments) was a strong predictor of SCD (HR 4.51, 95% CI 2.39–8.53).^134^ In another study, individuals with an acute myocarditis presentation (*n* = 97) and desmosomal gene variants (*n* = 36) were shown to have more LGE segments and higher ventricular arrhythmia compared with myocarditis patients without a desmosomal variant.^135^ Other conditions in which LGE has been shown to have prognostic role in sudden death or ventricular arrhythmia include neuromuscular disorders,^136^ Chagas disease,^137^ and arrhythmogenic cardiomyopathy.^138^

## Interstitial fibrosis and risk of sudden death

In addition to replacement fibrosis, there is also evidence to suggest interstitial fibrosis also has a role in the generation and maintenance of malignant arrhythmias and therefore SCD.^139^ Using T1 mapping on CMR, the longitudinal relaxation time of tissues is mapped on a pixel-wise map allowing quantification of myocardial tissue characteristics. This enables assessment of diffuse myocardial changes, in particular fibrosis.^140,141^ A number of studies have shown an association of native T1 values on CMR with SCD in both ischaemic and non-ischaemic cardiomyopathies.^139,142–145^ In patients with DCM and no evidence of LGE undergoing catheter ablation of VT, diffuse fibrosis estimated by using post-contrast T1 mapping was found to correlate with the unipolar > bipolar voltage abnormality at electroanatomic mapping and shorter post-contrast T1 time was associated with an increased risk of VT recurrence.^146^ Whilst the body of evidence is growing and compelling, there remains some uncertainty as to whether T1 mapping provides additional value to LGE in SCD prediction. Further studies are also required to ascertain if T1 mapping is superior to extracellular volume measurement.^147,148^

## LGE to guide ablation of persistent ventricular tachycardia

CMR, particularly with LGE, has established itself as a cornerstone in non-invasive VT substrate identification, providing crucial information on dense scar tissue and the adjacent ‘border zones’, which often contain VT isthmuses.^149,150^ Radiofrequency catheter ablation is an established treatment method for VT and has been shown to reduce ICD therapy and VT burden by 50%–75%.^151^

In patients with DCM, pre-ablation procedure imaging to identify the anticipated location of the VT substrate and the typical midseptal vs. LV free wall lateral and frequently sub-epicardial LGE is routinely performed to optimize procedural planning and approach to electroanatomic mapping.^152^ Integration of CMR with electroanatomic mapping systems presents a robust approach for guiding catheter ablation procedures, allowing for comprehensive assessment of scar-related VT substrates. Studies have shown LGE-guided elimination of critical VT isthmus sites predicted by the presence of imaging defined channels or corridors and have suggested comparable VT ablation outcome even without induction and mapping of the tachycardia,^150,153,154^ thereby underpinning the value of LGE guidance in identifying and targeting VT substrates particularly in the setting of CAD. Software advances allow for detailed scar segmentation and identification of three-dimensional conducting channels or corridors that can facilitate targeting of potential VT isthmus sites from adjacent endocardial and/or epicardial sites. VOYAGE (ClinicalTrials.gov NCT04694079) is a multi-centre, randomized controlled trial designed to compare CMR-guided VT ablation with the standard electroanatomic mapping-guided approach with respect to procedural and 12-month follow-up outcome, assessing efficacy, safety, and procedural duration.^155^

## LGE and randomized controlled trial evidence

Current guideline recommendations for using CMR-LGE rely on observational data. Whilst risk scores exist to guide ICD implant decisions, these have not been tested prospectively in randomized controlled trials. However, a number of randomized controlled trials are currently recruiting to bridge this gap in evidence. The PROFID consortium is currently recruiting for the PROFID EHRA trial (ClinicalTrials.gov NCT05665608), which will seek to randomize 3595 patients with ischaemic heart disease into ICD plus contemporary medical therapy vs. medical therapy alone.^156^

Supported by a large body of observational data suggesting a strong correlation between LGE and SCD events in DCM, two randomized controlled trials, BRITISH (NCT05568069)^157^ and CMR-GUIDE (NCT01918215),^158^ are currently underway to evaluate LGE-guided ICD implantation. The results of these trials may prove crucial in pivoting away from LVEF based risk stratification when it comes to offering ICD therapy in DCM.

## Guidelines recommendations for LGE in sudden death risk stratification

Both European and American guidelines have recommendations to incorporate LGE in aiding diagnosis and risk stratification. In the most recent ESC cardiomyopathy management guidelines, in intermediate risk category patients with HCM, extensive LGE (≥15% of LV mass) has been recommended as a consideration in offering prophylactic ICD implant with a class IIb recommendation, acknowledging the lack of robust data, the variability and lack of consensus in quantification methods (level of evidence B).^42^ Similarly, ‘extensive’ LGE, in the absence of other high-risk features, carries a level 2b recommendation for primary ICD implant in the AHA/ACC guidelines for the management of HCM.^41^ For DCM, broadly the guideline recommendations for ICD implants are based around the ejection fraction. However, in those patients without severe LV impairment, the European guidelines recommend offering an ICD (if LVEF is below 50%) when LGE is present in addition to one other major risk factor (class IIa),^2^ which downgrades to a class IIb (level of evidence C) when no other high-risk features are present.^42^ These guidelines are likely to evolve given ongoing randomized controlled trials testing LGE-guided interventions.

## Future advances

The field of radiomics assessing shape and texture features derived from CMR scans including LGE heterogeneity appears promising with incremental utility to existing risk scores for SCD although still warrants further validation.^159^

Computational *in silico* modelling and artificial intelligence is transforming many aspects of biomedicine, with cardiology being a particular area of focus.^160,161^ Whilst LGE-CMR provides important anatomical information regarding tissue remodelling and fibrosis deposition, personalized (‘digital twin’) *in silico* modelling approaches have the ability to probe the *functional* consequences of such *structural* changes. Most often reconstructed directly from LGE-CMR data, these personalized image-based models are typically combined with virtual induction stimulation protocols to attempt to assess patient-specific arrhythmia vulnerability.

**Figure 1:**
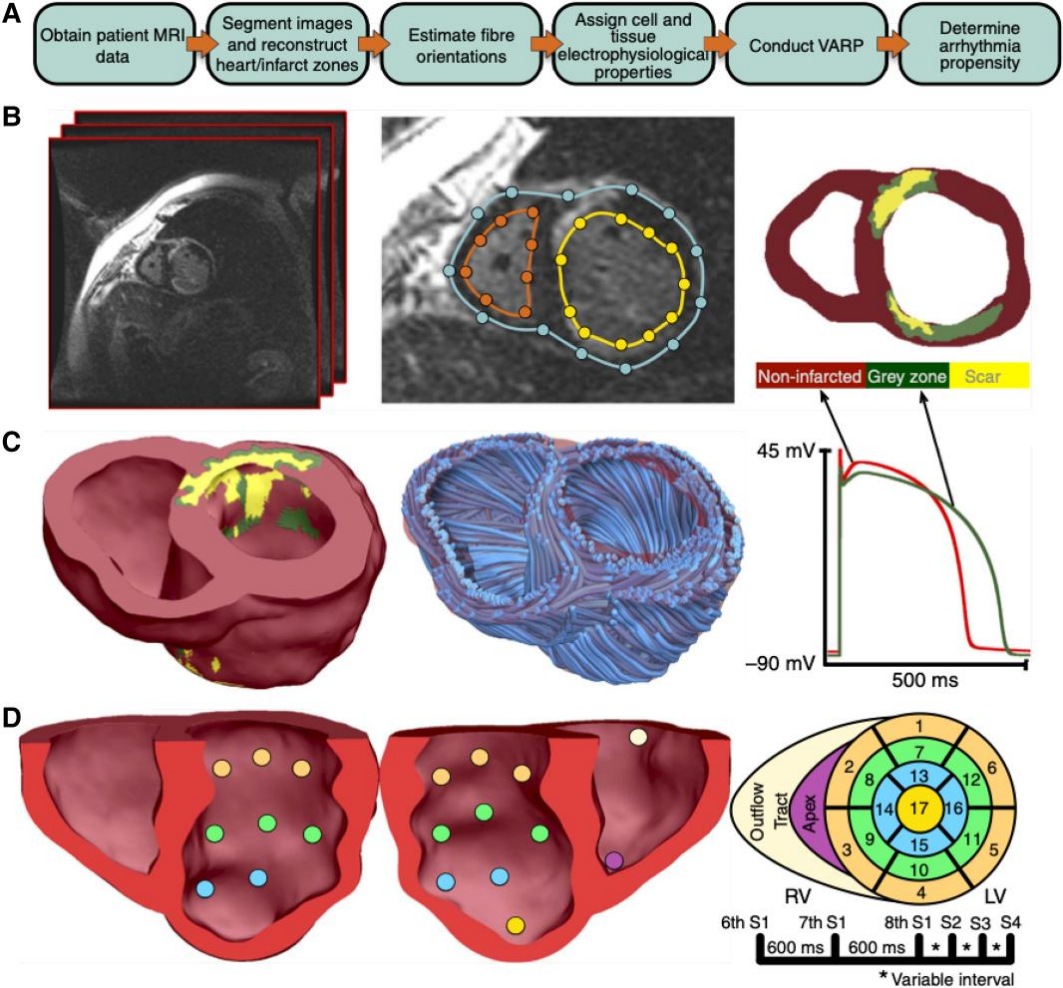
Original figure from Arevalo *et al*.^162^ showing the procedure of creating patient-specific anatomical models from late gadolinium enhancement (LGE)-cardiac magnetic resonance (CMR) data and conducting a virtual stimulation induction protocol to uncover arrhythmia vulnerability

In the original study from Arevalo *et al.*,^162^ personalized models were created from 41 myocardial infarction ICD recipients (*Figure 1*). Application of a subsequent *in silico* assessment of arrhythmic risk (as a binary metric) was shown to be strongly associated with ICD events in follow-up (HR 4.05, 95% CI 1.20–13.8; *P* = .03), outperforming conventional clinical metrics such as LVEF (HR 0.95, 95% CI 0.90–1.01; *P* = .12) as well as image-based metrics such as scar volume (HR 1.02, 95% CI 0.99–1.04; *P* = .16). This approach was later interestingly adapted in a smaller HCM cohort (*n* = 26); whereby, LGE information was combined with T1 mapping to represent both focal and diffuse fibrosis within the personalized models.^163^ Here, the virtual induction protocol successfully categorized patients with ventricular arrhythmias achieving sensitivity 84.6%, specificity 76.9%, and accuracy 80.1%, outperforming current clinical risk predictors. Zhao *et al.* have developed a machine learning (ML) approach integrating CMR imaging and clinical characteristics in 758 patients with HCM showing that such a model outperformed the classic HCM Risk-SCD model with an improvement of 22.7% in the area under the curve (AUC).^164^ Shade *et al.*^165^ later demonstrated the utility of combining simulation-derived metrics from the induced arrhythmias in personalized models with both clinical metrics and imaging biomarkers within a supervised ML classifier. In a cohort of 45 cardiac sarcoidosis patients, they showed an AUC of 0.754 in the receiver operating characteristic analysis for this combined simulation-ML approach, outperforming clinical metrics alone. An alternative use of simulations in this context was presented by Balaban *et al.*^166^ in a much larger cohort of 156 non-ischaemic DCM patients. Here, the authors showed, not only could simulation outcomes themselves be used directly to confer arrhythmic risk (HR 1.40, 95% CI 1.23–1.59; *P* < .01), but simulations also demonstrated valuable mechanistic insight to help explain the uncovered association of the novel LGE biomarker scar-interface length (HR 1.75, 95% CI 1.24–2.47; *P* = .001) with arrhythmia occurrence.

These early studies demonstrated the exciting possibility of using digital twin CMR-derived computational models, including reconstructed scar anatomy from LGE data and mean entropy from T1 mapping data, to predict arrhythmia risk in a range of cardiomyopathies. Applications to significantly larger cohorts, along with rigorous external validation, is now imperative in order to galvanize clinical confidence in these digital approaches.^167^ However, with such virtual protocols taking 1–2 days per patient on a specialized high-performance computing facility, such analysis of larger cohorts necessitates the use of novel near real-time simulation tools to enable practical computation of results.^168^ Such novel approaches are based on topological path-finding algorithms which attempt to uncover potential pro-arrhythmic circuits through the 3D reconstructed scar substrate within LGE-based models. Early studies, albeit in small cohorts (<40) have shown utility of such a near real-time approach in identifying VT recurrence following ablation^169^ and appropriate ICD therapy.^170^

One primary limitation of any biomarker for risk assessment based on LGE segmentations, or indeed a computational model derived from these images, is the necessary choice of segmentation method, along with possible thresholds or associated parameters that define the binary LGE segmented image, which have been shown to directly affect risk prediction.^171^ Whilst such choices are far from standardized, some studies have indeed demonstrated consistent findings independent of the segmentation method used.^30^

**Figure 2:**
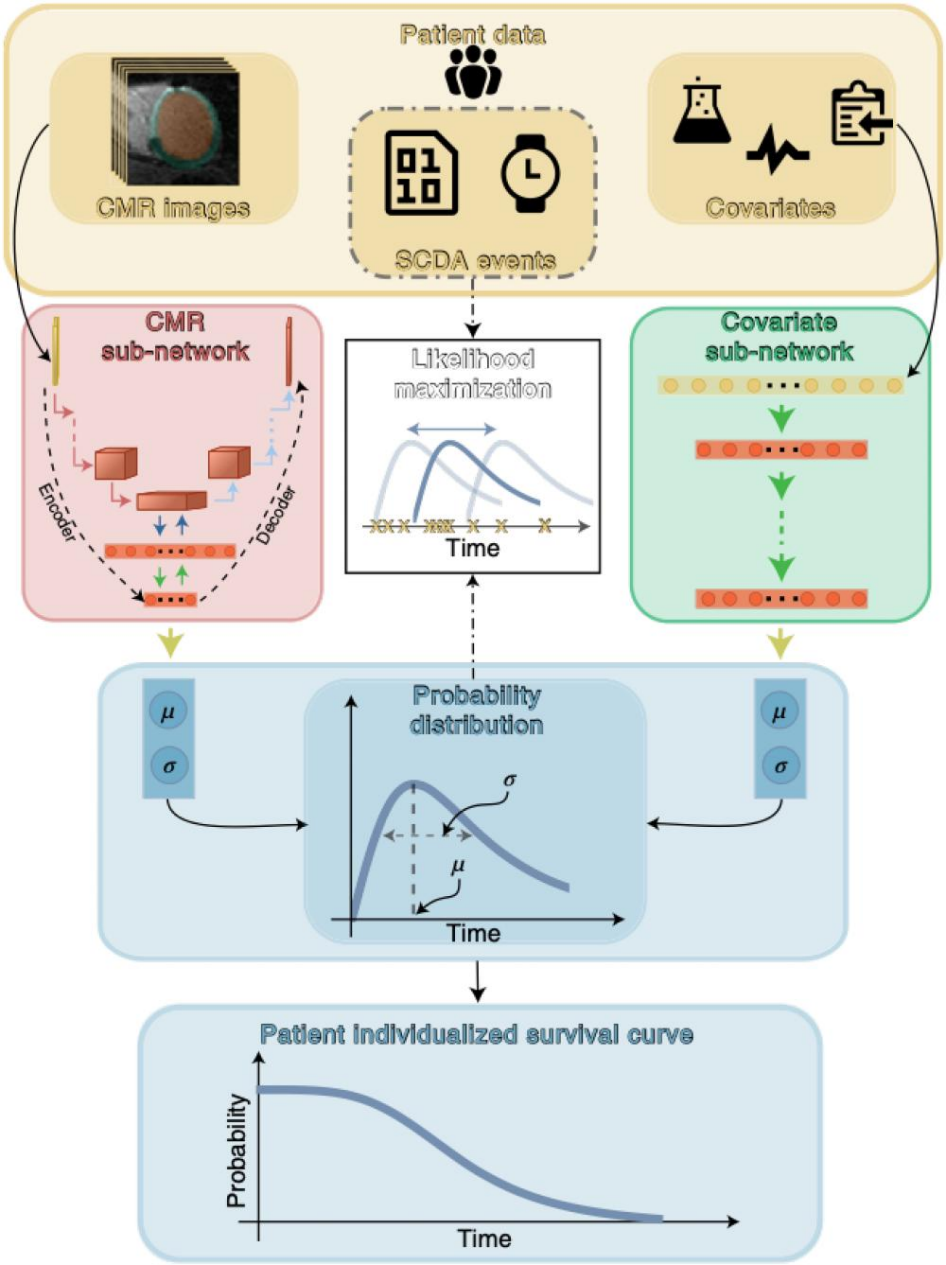
Procedure for generating deep learning (DL) predicted survival curves proposed by Popescu *et al*. (unedited figure)^172^ by integrating DL networks trained on raw late gadolinium enhancement (LGE) images with other clinical covariates

However, in a recent study by Popescu *et al.*,^172^ they attempted to remove the segmentation process entirely, instead training a deep learning (DL) model directly on the raw LGE images themselves, with the addition of clinical covariates (*Figure 2*). The DL-predicted survival curves, which included uncertainty, outperformed standard approaches. As greater volumes of training data become increasingly available, DL-approaches such as these will likely increase further in their accuracy. It is likely that these methods, potentially augmented by digital twin simulations, are integrated into clinical workflows to risk stratify patients.

## Conclusion and areas of unmet need

LGE-enabled tissue characterization has transformed clinical practice in the last 30 years. A formidable body of evidence shows a strong association of LGE with malignant arrhythmia and SCD in both ischaemic and non-ischaemic cardiomyopathies. Current guidelines advocate the use of LGE CMR for risk stratification, particularly in intermediate risk cases to guide life altering therapy decisions. However, challenges remain. Most studies lack broad demographic representation and no consensus exists in the current quantification methods. In addition, many stratification risk scores for SCD were developed historically based on clinical parameters, excluding modern imaging techniques. There is the unmet need to develop robust risk scoring algorithms derived and validated in multi-ancestry populations including under represented cohorts such as children, women and elderly patients. Despite the update in the guidelines, randomized controlled trials have yet to validate the use of a modern risk scoring system that incorporates LGE assessment on CMR to guide therapy decisions, although ongoing trials are seeking to bridge this gap in knowledge. Lastly, the inexorable rise of ML and digital twin technologies present unique opportunities to further transform the field and patient lives.

## Declarations

### Data Availability

No data were generated or analysed for or in support of this paper.
